# Unusual Switch of Cell Death Mode by Docosahexaenoic Acid (DHA) via Enhancing Endoplasmic Reticulum Peroxidation Induced by Lipophilic Iron Complex

**DOI:** 10.3390/antiox15101303

**Published:** 2026-10-09

**Authors:** Bo Shao, Zhi-Guo Sheng, Huan Li, Shun-Li Jiang, Gui-Zhi Han, Chen-Jun Shao, Ben-Zhan Zhu

**Affiliations:** 1 School of Public Health, Jining Medical University, Jining 272013, China; 2 State Key Laboratory of Environmental Chemistry and Ecotoxicology, Research Center for Eco-Environmental Sciences, Chinese Academy of Sciences, Beijing 100085, China; 3 School of Resources and Environment, The University of Chinese Academy of Sciences, Beijing 100085, China; 4 Linus Pauling Institute, Oregon State University, Corvallis, OR 97331, USA

**Keywords:** ferroptosis, 8-hydroxyquinoline, lipophilic iron complexes, endoplasmic reticulum, mitochondrial

## Abstract

Although ferroptosis is a programmed iron-dependent cell death, many lipophilic iron complexes fail to induce ferroptosis through iron overload. The underlying mechanisms remain unclear. Here we found that a typical lipophilic iron complex 8-hydroxyquinoline (8HQ)-Fe(III) (1: 1, 10 μM), can efficiently induce cellular iron overload, lipid peroxidation, and finally cytotoxicity. However, the resulting cell death mode was not typical ferroptosis: it was not inhibited by the classic ferroptosis inhibitors and exhibited mitochondrial swelling rather than shrinkage. Mechanistic studies revealed that 8HQ-Fe(III) triggered both endoplasmic reticulum (ER) peroxidation and mitochondrial damage. The ER-targeted antioxidant (stobadine) inhibited lipid peroxidation but did not suppress cell death. In contrast, mitochondrial protectants effectively blocked 8HQ-Fe-induced cell death by mitigating mitochondrial damage. This indicates that mitochondrial damage plays an overwhelmingly dominant role in driving cell death. Strikingly, pre-incubation with docosahexaenoic acid (DHA) can strongly enrich the ER membrane and increase its peroxidation. As expected, both stobadine and the well-known ferroptosis inhibitor (Fer-1) suppressed 8HQ-Fe(III)-induced cell death via inhibition of ER peroxidation, while mitochondrial protectants failed to prevent cell death. Mitochondrial shrinkage was also observed. Thus, making ER peroxidation the dominant driver of cell death by DHA pretreatment will switch the cell death modality from non-ferroptosis to classical ferroptosis. This study revealed that the balance between ER peroxidation and mitochondrial damage dictated the type of iron-dependent cell death, and established DHA-primed 8HQ-Fe(III) as a novel, tunable strategy for efficiently inducing ferroptosis by deliberately shifting subcellular peroxidation dominance.

## 1. Introduction

Ferroptosis, a form of cell death that depends on iron, triggered by lipid peroxidation (LPO), holds promise for treating conditions such as cancer and degenerative disorders, despite the fact that the process still cannot be fully understood [1,2]. Over the last decade, and especially in the last few years, considerable advances have been achieved in deciphering how ferroptosis is regulated. This progress has, in turn, elevated the importance of ferroptosis for biological and medical research.

Advancements in the induction of ferroptosis are important for gaining deeper insights into the cell death mechanisms and for investigating ferroptosis-related diseases. Initiating ferroptosis through pharmacological or genetic interventions has been reported to slow down and reverse tumor proliferation in cancer-affected organisms [3,4,5]. Moreover, the induction of ferroptosis can be employed to construct models of this process in both in vivo and in vitro settings, enabling more in-depth studies of its underlying mechanisms [6,7,8]. Most recent studies on ferroptosis induction focused on inhibiting cystine transport via system xc^−^ or forming a covalent bond with the selenocysteine at the active site of glutathione peroxidase 4 (GPX4). These approaches caused GPX4 inactivation and raised the levels of endogenous lipid ROS whose generation relies on iron [7,9,10]. As a type of iron-dependent programmed cell death executed through LPO, direct stimulation of exogenous iron overload and subsequent LPO were believed to be the most direct and readily available method for inducing ferroptosis. However, related research remains scarce. Although some studies have indicated that ferric ammonium citrate (FAC) can induce ferroptosis through iron overload, the induction efficiency is low [11,12].

The development of efficient iron overload-based ferroptosis inducers offers several key advantages. First, excessive iron accumulation plays a pivotal role in certain diseases, such as hereditary hemochromatosis [13], brain trauma [14], certain neurodegenerative diseases [12,15], alcoholic liver disease [16], and intervertebral disc degeneration [11]. The development of efficient ferroptosis inducers via iron overload may better simulate these disease mechanisms in vitro and in vivo, facilitating further research on iron overload and ferroptosis-related diseases. Furthermore, effective induction of ferroptosis holds significant promise as an approach for treating certain forms of cancer [17,18,19]. During our previous investigations into the chemical and biomedical effects induced by metal complexes [20,21,22,23,24,25,26,27,28,29,30,31], we have placed significant emphasis on translocating various metal complexes across the cell membrane by forming hydrophobic binary/ternary complexes or ion-pairing associates. Thus, we asked whether a small-molecule iron chelator could efficiently promote iron uptake into cells by forming a lipophilic iron chelate, consequently triggering ferroptosis.

Recent evidence indicates that ferroptosis does not represent a single, uniform form of cell death, but rather a complex event involving multiple subcellular compartments, among which the endoplasmic reticulum (ER) and mitochondria (Mito) play central yet distinct roles [32,33]. Within eukaryotic cells, the ER constitutes the largest membrane-bound organelle and is responsible for synthesizing most membrane lipids, including PUFAs, a class of fatty acids with multiple double bonds that are prone to oxidation [34,35]. Consequently, the ER membrane is a major site for LPO. Studies have also indicated that ER peroxidation serves as a key step during ferroptosis, and aberrant activity of ER-resident proteins (such as GPX4 and certain iron metabolism-related enzymes) can directly trigger or exacerbate ferroptosis [36]. On the other hand, Mito serves as a center for iron utilization and storage; mitochondrial iron dyshomeostasis and dysfunction are also closely associated with ferroptosis [37,38]. The hallmark ultrastructural changes of ferroptosis, namely mitochondrial shrinkage and reduced cristae, highlight the crucial function of mitochondria in this death process [7]. However, the relative contributions of ER peroxidation versus mitochondrial damage to ferroptotic outcomes remain unclear under different contexts.

8-Hydroxyquinoline (8HQ) is a classic lipophilic metal chelator known for its ability to form stable, redox-active complexes with transition metal ions [39]. Previous studies have demonstrated that 8HQ-Fe(III) complexes can efficiently deliver iron into cells and induce iron-dependent cell death [40]. However, whether this cell death corresponds to ferroptosis remains incompletely understood. A previous study by Fang et al. reported that 8HQ-Fe(III)-induced cell death could not be inhibited by ferrostatin-1 (Fer-1) or liproxstatin-1, suggesting it is not ferroptosis; nevertheless, the underlying mechanism was not elucidated, and whether this death modality could be switched to ferroptosis by manipulating subcellular oxidative damage had not been explored [41].

In this study, we unexpectedly found that 8HQ-Fe(III) rapidly and efficiently induced iron overload and LPO. Mechanistically, although 8HQ-Fe(III) triggered both ER peroxidation and mitochondrial damage, the latter played the dominant role in driving cell death. Strikingly, pre-incubation with docosahexaenoic acid (DHA) enriched ER membranes and enhanced ER peroxidation, thereby completely switching the iron-dependent non-ferroptotic cell death to classical ferroptosis. A shift in the dominant driver from mitochondrial damage to ER peroxidation switches the mode of cell death.

These results not only provided a proof-of-concept that manipulating subcellular peroxidation patterns can switch cell death modalities, but also introduced a novel, highly efficient, and mechanistically defined system, namely 8HQ/Fe(III) combined with DHA, for inducing ferroptosis. Compared to conventional ferroptosis inducers (e.g., erastin, RSL3, FINO2), this system offered rapid action (within hours), low concentrations (10 μM), and cost-effectiveness. It was assumed that this approach held significant promise for developing ferroptosis-inducing drugs and establishing in vitro and in vivo models that faithfully recapitulated iron overload-driven ferroptosis, thereby facilitating deeper mechanistic studies and therapeutic interventions for ferroptosis-related diseases.

## 2. Materials and Methods

### 2.1. Cell Line

HT-1080 (human (*Homo sapiens*) male fibrosarcoma) cells were obtained from the Cell Resource Center, IBMS, CAMS/PUMC. The accession number is 1101HUM-PUMC000070 (National Infrastructure for Cell Resource).

### 2.2. Chemicals and Reagents

Reagents used in this study included: 8HQ (MedChemExpress), FeCl_3_ (Sinopharm Chemical Reagent), Calcein (Aladdin), Ferrostatin-1 (Fer-1) (MedChemExpress), Lip-1 (MedChemExpress), Trolox (Sigma), DIP (Sigma), Deferoxamine (DFO) (Sigma), cck-8 (Dojindo), FerroOrange (Dojindo), Calcein-AM (Dojindo), C11 BODIPY^581/591^ (MedChemExpress), Liperfluo (Dojindo), ER tracker Blue-white (GLPBIO), Mito tracker Deep Red (Beyotime Biotechnology), Lyso Deep Red (Invitrogen), JC-1 kit (Beyotime Biotechnology), mPTP kit (Beyotime Biotechnology), docosahexaenoic acid (DHA) (MedChemExpress).

### 2.3. Cell Viability Assays

The cells were exposed to 8HQ-Fe(III) (10 μM, 1:1) at 37 °C under 5% CO_2_ for 2 h. For experiments involving DHA (10 μM), cells were pre-treated for 24 h, then washed. Cell viability was assessed using a CCK-8 kit (Promega), and the data were analyzed using GraphPad Prism 9. Each treatment was repeated 6 times. In all experiments, untreated cells that received only an equal volume of solvent (DMSO or PBS) without 8HQ-Fe(III) or other treatments were used as the control.

### 2.4. Quantification of Iron Concentration via Inductively Coupled Plasma-Mass Spectrometry

Cells (0.5 × 10^6^ cells/mL) were exposed to varying ratios of 8HQ-Fe(III) for 30 min. They were then counted and subjected to overnight digestion at ambient temperature using 60% HNO_3_. Iron content was then measured via ICP-MS. Each treatment was carried out in triplicate.

### 2.5. Lipid Peroxidation Analysis

Using C11 BODIPY581/591 as a probe, cells (10^4^) were incubated with Fe(III) or 8HQ-Fe(III) for 15 min at 37 °C. They were then treated with the C11 BODIPY581/591 probe (1 μM) for 30 min. The fluorescence signals from both the oxidized and reduced forms of the probe were visualized using a Leica TCS SP CLSM (excitation: 488/561 nm; emission: 510–550/600–650 nm). For quantitative analysis, 20 cells from each group were randomly selected across different microscopic fields. The fluorescence of the oxidized form of C11 BODIPY581/591 was also assessed via flow cytometry.

For our Liperfluo assay, cells (10^6^ cells/mL) were incubated with Liperfluo (1 μM) for 15 min at 37 °C. They were then treated with Fe(III) or 8HQ-Fe(III) for 15 min. Fluorescence was quantified via flow cytometry (BD FACS Caliber) with excitation at 488 nm and emission at 515 nm. All treatments were conducted in triplicate.

The analysis of 4-HNE was conducted following standard procedures using commercially available diagnostic kits (4-HNE assay kit [Abcam, ab238538]).

### 2.6. Electron Spin Resonance (ESR) Spin-Trapping Studies

We used Fe(III), AA, and H_2_O_2_, together with DMPO (100 mM) as the spin trap, in a 100-mM ammonium acetate buffer (pH 7.4), with or without iron complexes. ESR measurements were performed at ambient temperature using a Bruker ESR 300 spectrometer. For specific parameters, please refer to the relevant literature [42].

### 2.7. Fluorescence Competition Assay with Calcein

Calcein-containing solutions, comprising various concentrations of chelators and Fe(III), were used for a fluorescence assay within a 20 mM HEPES buffer (pH 7.4). The fluorescence detection was performed at an excitation wavelength of 488 nm and emission at 515 nm. The recorded fluorescence spectra were captured using a Cary Eclipse fluorescence spectrophotometer (Varian, Palo Alto, CA, USA).

### 2.8. Fourier Transform Ion Cyclotron Resonance Mass (FT-ICR-MS) Detection 

Multiple reaction mixtures were prepared, spanning 100 μM of 8HQ and 10 μM of Fe(III) in 20 mM ammonium acetate buffer at pH 7.4. The complex of 8HQ-Fe(III) was identified using FT-ICR-MS. (Bruker Daltonik, GmbH, Bremen, Germany). For specific parameters, please refer to the relevant literature [42].

### 2.9. Labile Iron Pool Determination 

Cells (10^6^ cells/mL) were incubated with the calcein-AM probe (0.5 μM) for 15 min at 37 °C. They were then exposed to Fe(III) or 8HQ-Fe(III) (10 μM, 1:1) for 10 min. Fluorescence was then quantified using flow cytometry (BD FACS Caliber, San Jose, CA, USA) at 488 nm excitation and 515 nm emission.

For FerroOrange assays, cells (10^6^ cells/mL) were incubated with Fe(III) or 8HQ-Fe(III) (10 μM, 1:1) for 20 min at 37 °C. They were then treated with the FerroOrange probe (1 μM) for 30 min. Fluorescence was visualized using a CLSM (Leica TCS SP, Wetzlar, Germany) at 561 nm excitation and 650 nm emission.

### 2.10. Ultraviolet-Visible Spectra Assay

Multiple reaction mixtures of 0.5 mL volumes were prepared, spanning varying concentrations of 8HQ and Fe(III) in 100 mM phosphate buffer (PB) solution at pH 7.4, or in 1-octanol for spectral examination. Their UV-vis spectra were recorded using a Cary 3500 UV-vis spectrophotometer (Agilent, Santa Clara, CA, USA) (3).

### 2.11. 1-Octanol-Water Partition Assay

The partitioning behavior of solutions of 8HQ-Fe(III) complexes of varying concentrations between organic (1-octanol) and aqueous phases was investigated via the shake-flask method. The concentration of iron within distinct phases was quantified through ICP-MS using a NexION 300X instrument (PerkinElmer, Waltham, MA, USA).

### 2.12. Density Functional Theory Computational Analysis

To assess the Fe(III) binding ability of the ligand, we used a DFT computational analysis. The structural optimizations of the ligands and their Fe(III) complexes were carried out via the B3LYP method. The calculations used a composite basis set consisting of LANL2DZ for iron and 6-31+G (d,p) for nonmetal elements, as detailed. For the computation of thermodynamic parameters, frequency analyses were performed at the same theoretical level. Considering the solvent effects, a single-point energy calculation was performed on the optimized structures using the B3LYP method, with the following basis sets: 6-311++G (d,p) for nonmetal elements, the def2TZVP basis set for iron, and the PCM solvation model. All computations were done using Gaussian 09 software. The visual representation of complex structures was done on CYLview (www.cylview.org, accessed on 28 July 2023). The binding energy of each Fe(III) complex was measured relative to the 8HQ-Fe(III) (1:1) complex, with the binding energy unit expressed in kcal/mol.

### 2.13. Fluorescent Staining of Live Cells

Cells (10^6^ cells/mL) were incubated with Fe(III) or 8HQ-Fe(III) (10 μM, 1:1) for 15 min at 37 °C. Fluorescent labels were employed following the instructions provided by their respective manufacturers. ER tracker blue-white (Ex: 405 nm, Em: 430–500 nm) (GLPBIO), Mito tracker Deep Red (Ex: 644 nm, Em: 660–750 nm) (Invitrogen), and LysoTracker Deep Red (Ex: 644 nm, Em: 660–750 nm) (Invitrogen) were diluted to a concentration of 50 nM in HBSS and then incubated with the cells for 30 min at 37 °C. C11 BODIPY^581/591^ (Oxidized: Ex: 488 nm, 510–550 nm; Reduced: Ex: 561 nm, Em: 600–630 nm) was diluted to a concentration of 1 µM and similarly incubated with the cells for 30 min at 37 °C.

### 2.14. Multi-Structured Illumination Microscopy Fluorescence Imaging

Cells (10^6^ cells/mL) were incubated with Fe(III) or 8HQ-Fe(III) (10 μM, 1:1) for 15 min at 37 °C. Multi-structured illumination microscopy (Multi-SIM) images of HT-1080 cells were captured using the Multi-SIM imaging system (NanoInsights-Tech, Beijing, China). For specific parameters, please refer to the relevant literature [43].

### 2.15. Statistical Analysis

Statistical analysis was conducted using Prism 9.0.0. A one-way ANOVA was initially applied, followed by a least significant difference (LSD) test to ascertain significant distinctions among various treatment groups.

## 3. Results

Iron overload was critical in ferroptosis, and inducing ferroptosis through direct iron overload was of great significance for the research of ferroptosis-related diseases. Currently, the commonly used method to induce ferroptosis via iron overload was pre-incubation with ferric ammonium citrate (FAC). However, this method required a high concentration of FAC (5 mM) and a long incubation time (72 h), thus resulting in low efficiency in inducing ferroptosis. Forming lipophilic iron complexes to enhance cellular iron uptake efficiency may represent a more effective method for inducing ferroptosis through iron overload. However, few breakthroughs have been achieved in this area. The main reason was that cell death induced by the formation of lipophilic iron complexes, despite its high efficiency, often did not exhibit characteristic features of ferroptosis. As a highly lipophilic iron chelator, 8HQ was used in this study to explore whether it can efficiently induce ferroptosis by forming lipophilic iron complexes with iron.

### 3.1. 8HQ-Fe(III) Induced Iron-Dependent but Non-Ferroptotic Cell Death by Forming a Lipophilic Iron Complex

To evaluate cell death, we initially used the CCK-8 assay, which measures cellular metabolic activity. While this assay does not directly quantify cell death, it is widely used in the ferroptosis field for initial screening and quantitative assessment of cell viability [44,45,46]. To support our findings, we corroborated the CCK-8 results with additional evidence, including TEM morphology and lipid peroxidation markers, as described below.

**Figure 1:**
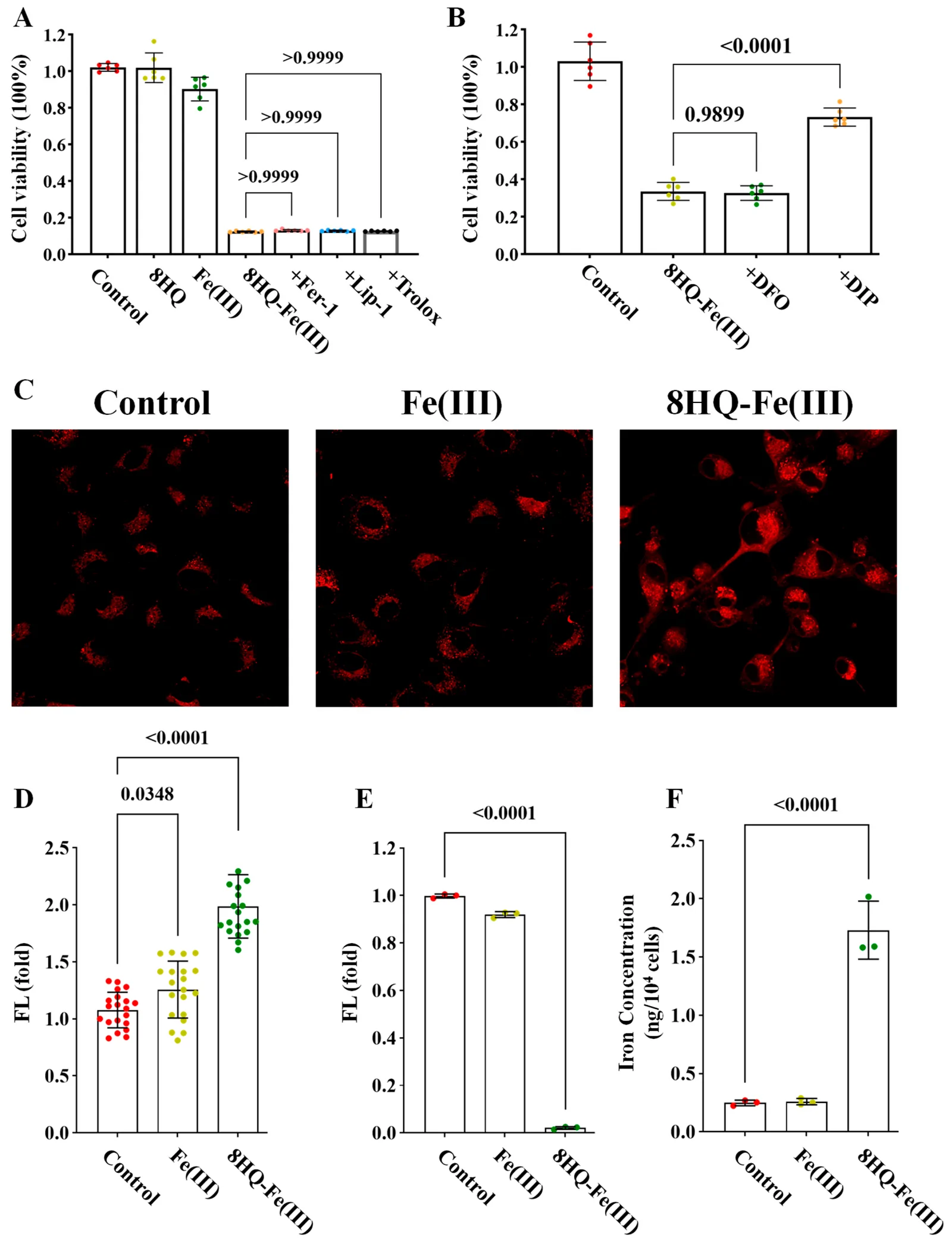
**8HQ-Fe(III) induced iron-dependent cell death in HT-1080 cells.** (**A**) Rescue of HT-1080 cells treated with 8HQ-Fe(III) (10 μM, 1:1) for 2 h by Fer-1, Lip-1 and trolox. Cell viability was measured by CCK-8 assay. Data are represented as mean ± SD, n  =  6 biologically independent samples. (**B**) Rescue of HT-1080 cells treated with 8HQ-Fe(III) by 200 μM DFO or 10 μM DIP. DFO and DIP were pre-incubated for 5 h, then washed away, followed by 8HQ-Fe(III) treatment (10 μM, 1:1, 2 h). Data are represented as mean ± SD, n  =  6 biologically independent samples. (**C**) Representative confocal microscopy images demonstrate intracellular LIP within cells treated with 8HQ-Fe(III) for 20 min, as evidenced by FerroOrange probes. The quantified fluorescence data are presented in (**D**), and the values are represented as mean ± standard deviation (SD), n  =  20. (**E**) The change in LIP level in the cells treated with 8HQ-Fe(III) for 10 min, as detected by Calcein-AM. Data are represented as mean ± SD, n  =  3. (**F**) Total iron concentration in cells treated with 8HQ-Fe(III) for 30 min, as detected by ICP-MS. Control, untreated cells. Data are represented as mean ± SD, n  =  3.

It was first found that treatment with either 10 μM 8HQ or 10 μM Fe(III) alone did not induce cell death, whereas the combined treatment of 8HQ and Fe(III) significantly induced cell death (Figure 1A). We further investigated the cell death induced by 8HQ-Fe(III) in the absence or presence of classic ferroptosis inhibitors such as Ferrostatin-1 (Fer-1), Liproxstatin-1 (Lip-1), and Trolox. As shown in Figure 1A, the cell death induced by 8HQ-Fe(III) could not be inhibited by Fer-1, Lip-1, and Trolox. In addition, apoptosis and necroptosis inhibitors such as Z-VAD-FMK, Necrostatin-1, GSK872, and Necrosulfonamide were also used to detect the cell death induced by the lipophilic iron complex. Figure S1 showed that neither apoptosis nor necroptosis was involved in the cell death (Figure S1). However, the lipophilic iron chelator DIP significantly inhibited cell death, whereas the hydrophilic iron chelator DFO did not (Figure 1B). These findings suggested that 8HQ-Fe(III)-induced cell death was closely associated with intracellular iron overload.

It has been suggested that the labile iron pool (LIP) facilitated redox cycling by functioning as an intermediary reservoir between extracellular iron and protein-associated intracellular iron. While the level of LIP can vary in response to stimuli, these fluctuations were generally transient. However, under conditions of excess or deficient iron levels, these variations may potentially disturb the cellular equilibrium and jeopardize its stability [47]. Furthermore, we detected the internal free iron content of HT-1080 cells treated with 8HQ-Fe(III). Confocal laser scanning microscopy (CLSM) (Figure 1C,D) revealed that FerroOrange fluorescence was markedly elevated following the addition of 8HQ-Fe(III) compared to control cells and cells exposed to Fe(III) alone, indicating the upregulation of the LIP. To assess LIP alterations after exposure to 8HQ-Fe(III) complexes, calcein acetoxymethyl ester (Calcein-AM) was employed. Calcein-AM was a fluorescence probe for detecting iron concentration, as its fluorescence is quenched upon interaction with iron. Greater fluorescence reduction indicated higher iron concentration. Using flow cytometry (Figure 1E), it was shown that t the concurrent addition of 8HQ and Fe(III) resulted in a marked reduction in calcein fluorescence, indicating that 8HQ facilitated the incorporation of extrinsic iron into HT-1080 cells and elevated the LIP within them. To assess the cellular assimilation of iron facilitated by 8HQ, ICP-MS was used to precisely quantify iron uptake. As shown in Figure 1F, treatment with 8HQ-Fe(III) significantly increased the cellular uptake of iron. Collectively, 8HQ was thus able to efficiently facilitate the cellular uptake of exogenous iron, thereby increasing the LIP.

To further determine the mechanism underlying the highly efficient iron delivery by 8HQ-Fe(III), the physicochemical properties of the 8HQ-Fe(III) complex and its cellular uptake pathway were characterized and analyzed.

**Figure 2:**
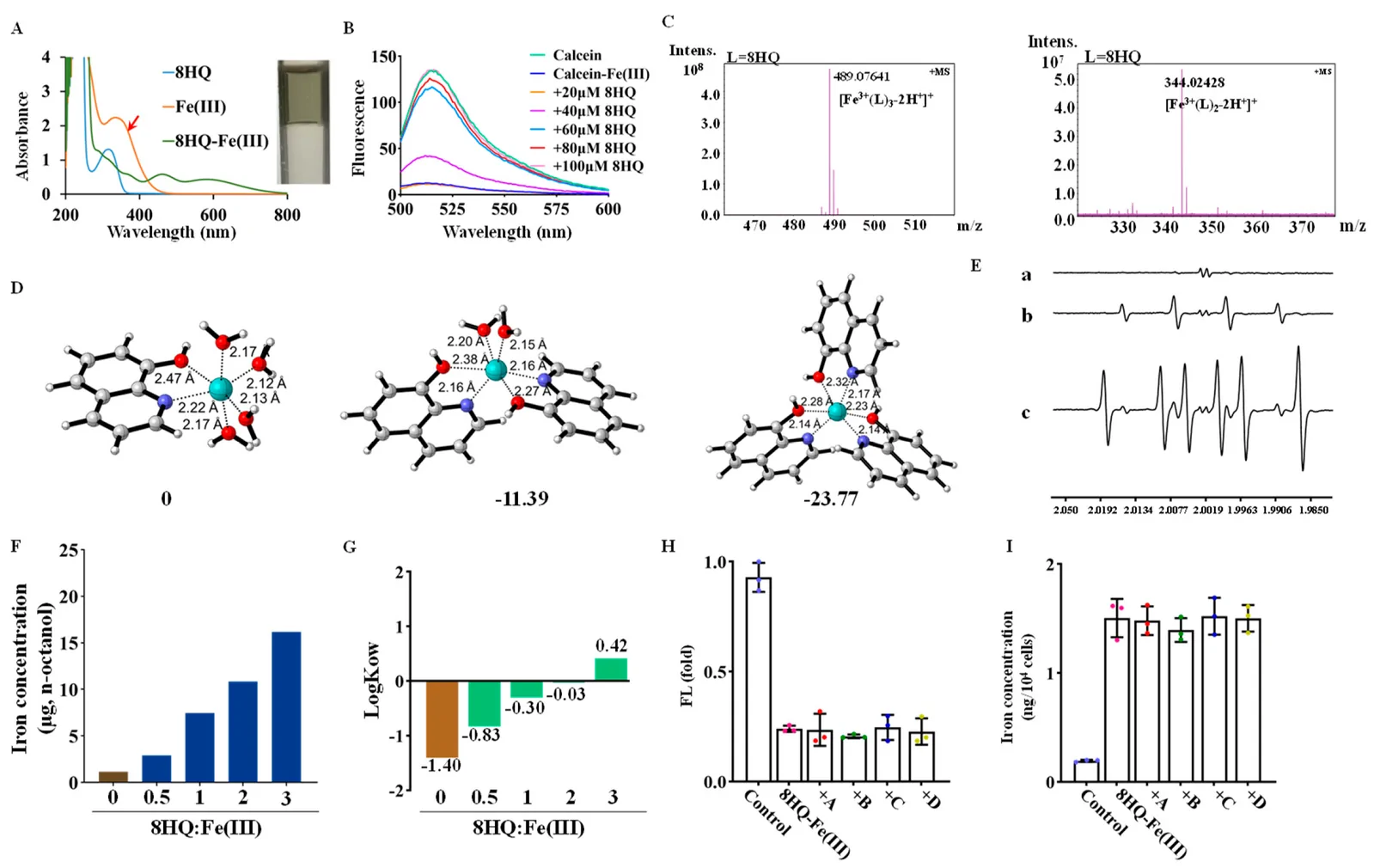
**Physicochemical characteristics of 8HQ binding Fe(III).** (**A**) UV-vis absorption spectra of 8HQ (10 μM) in the absence or presence of FeCl_3_ (10 μM). (**B**) Fluorescence quenching of calcein (0.5 μM) by FeCl_3_ (20 μM) in the absence or presence of 8HQ (20–100 μM) in 20 mM HEPES buffer (pH 7.4), measured at excitation/emission of 488/515 nm. (**C**) FT-ICR-MS spectrum of the reaction mixture containing 8HQ (50 μM) and Fe(III) (10 μM) in 20 mM ammonium acetate buffer (pH 7.4). (**D**) The proposed complex structure of 8HQ bound to Fe(III) (1:1–3:1), as calculated by DFT. The binding energy of Fe(III) complex is relative to 8HQ-Fe(III) (1:1) complex; the unit is kcal/mol. (**E**) ESR spectra of DMPO/•OH (•), DMPO/•CH_3_ (*) and Asc•− (¶) produced by incubation of DMPO with 8HQ-Fe(III)-AA-H_2_O_2_, with or without DMSO. (a) represents treatment of AA and H_2_O_2_; (b) represents treatment of 8HQ-Fe(III)-AA-H_2_O_2_; (c) represents treatment of 8HQ-Fe(III)-AA-H_2_O_2_ and DMSO. (**F**) Distribution of iron (10 μM) in n-octanol phases at different 8HQ:Fe(III) ratios (0.5:1 to 3:1), measured by ICP-MS. (**G**) Octanol/water partition coefficients (LogKow) of 8HQ-Fe(III) complexes at different ratios. Iron concentrations in n-octanol and water were analyzed by ICP-MS. (**H**) The cellular uptake of iron under the treatment of 10 μM 8HQ-Fe(III) in the presence of different inhibitors (10 mM 2-deoxy-D-glucose and 5 μM oligomycin, 10 μM colchicine, 10 μg/mL chlorpromazine, 500 μM cimetidine), using calcein-AM as probe. (**I**) Total iron concentration in cells treated with 8HQ-Fe(III) in the presence of different inhibitors, as detected by ICP-MS.

In Figure 2A, 8HQ rapidly binds Fe(III) to form iron complexes, which can be readily observed as an instantaneous alteration in color and ultraviolet-visible (UV-vis) spectra upon the introduction of Fe(III). The iron-binding affinity of 8HQ was assessed by calcein competition, revealing that 8HQ competed with calcein for iron binding (Figure 2B), with a calculated Kb(Fe(III)-8HQ) of approximately 1.03 × 10^22^ M^−1^. FT-ICR-MS indicated that 8HQ predominantly formed 3:1 and 2:1 complexes with iron (Figure 2C). DFT calculations showed that the binding energy of Fe(III) with 8HQ decreased as the 8HQ:Fe(III) ratio increased from 1:1 to 3:1, with the strongest binding at 3:1 (relative binding energy −23.77 kcal/mol) (Figure 2D).

Although 8HQ can form complexes with iron, the retention of redox activity by chelated iron was crucial for inducing cell death. ESR spin-trapping experiments demonstrated that the 8HQ-Fe(III) complex, in the presence of ascorbic acid (AA) and H_2_O_2_, generated spin-trapping agent DMPO/•OH and DMPO/•CH_3_ adducts, indicating Fenton-like reactivity occurred (Figure 2E). A DMPO/•OH-like signal was also observed with Fe(III) alone, but it could not be converted to DMPO/•CH_3_ in the presence of DMSO (Figure S2), consistent with nucleophilic addition to DMPO as previously reported [48].

The lipophilicity of the 8HQ-Fe(III) complex was a key determinant of its cellular uptake efficiency. As shown in Figure 2F, 8HQ promoted iron partitioning into the organic layer. The amount of Fe(III) complex within this layer grew as the 8HQ:Fe(III) ratio increased. The LogKow of the 8HQ-Fe(III) complex also increased as the ratio of 8HQ to Fe(III) increased (Figure 2G).

The routes by which 8HQ-Fe(III) enters cells were further studied. Cells were pretreated with metabolic inhibitors (2-deoxyglucose + oligomycin), endocytosis inhibitors (chlorpromazine, colchicine), and an organic cation transporters (OCT) inhibitor (cimetidine) [49]. However, none of these treatments reduced cellular iron uptake (Figure 2H,I), confirming that 8HQ-Fe(III) entered cells through energy-independent passive diffusion. Therefore, this passive diffusion route suggests that iron complexes can be widely applied across various cell types to serve distinct intracellular roles.

In summary, in the present study, 8HQ was suggested to rapidly chelate Fe(III) to form lipophilic, high-affinity complexes that retained redox activity and entered cells via energy-independent passive diffusion, thereby enabling efficient iron delivery.

### 3.2. 8HQ-Fe(III) Induced LPO and Mitochondrial Swelling but Not Ferroptotic Morphology

**Figure 3:**
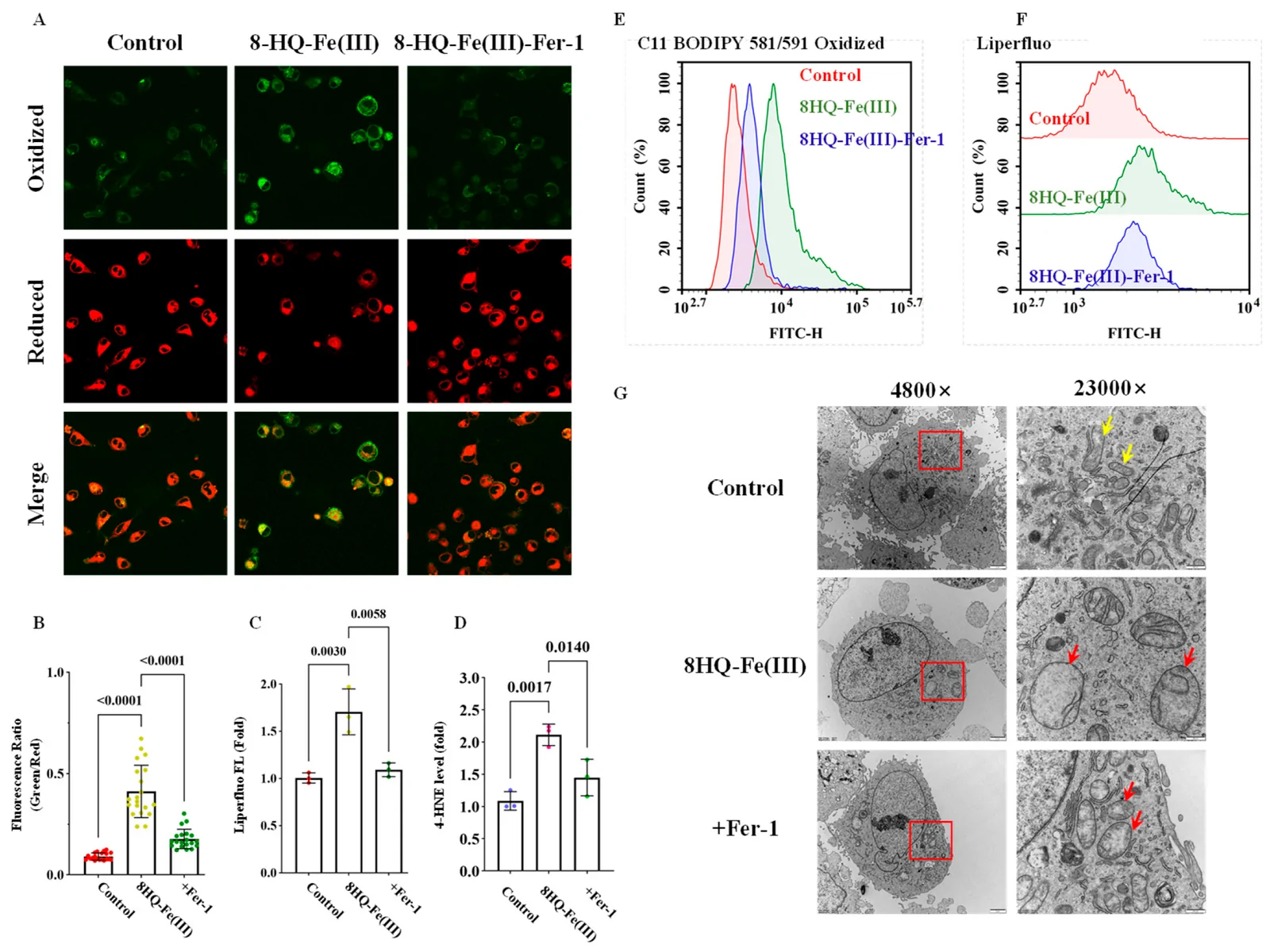
**LPO and morphological features of the cell death induced by 8HQ-Fe(III).** (**A**) 8HQ-Fe(III) (10 μM, 1:1, 15 min) induced Lipid ROS assessed by CLSM using C11-BODIPY^581/591^. The quantified fluorescence data are presented in (**B**), and the values are represented as mean ± standard deviation (SD), n  =  20. (**C**) Induction of LPO under treatment with 8HQ-Fe(III) (10 μM, 1:1, 15 min), as measured by FACS, using Liperfluo as probe. Data are represented as mean ± SD, n  =  3. (**D**) 4-HNE generation induced by 8HQ-Fe(III) (10 μM, 1:1, 30 min) in the absence or presence of Fer-1, as measured by Elisa kit. (**E**,**F**) Representative FACS images showing generation of lipid ROS in the cells treated with 8HQ-Fe(III), respectively, in the absence or presence of Fer-1, as reflected by C11-BODIPY^581/591^ probe (**E**) or Liperfluo probe (**F**). (**G**) Transmission electron microscopy of HT-1080 cells treated with 8HQ-Fe(III) (10 μM) for 1 h, in the absence or presence of Fer-1 (10 μM). Yellow arrow: normal mitochondria. Red arrow: damaged mitochondria. Control, untreated cells.

LPO, the process through which reactive oxygen species (ROS) react with lipids, resulted in the production of lipid hydroperoxides via the intermediate generation of peroxyl radicals. Ferroptosis was tightly linked to LPO and can be thought of as LPO-induced cell death. Thus, the C11-BODIPY^581/591^ reagent was then utilized to examine the generation of lipid ROS. As shown in Figure 3A,B,E, the generation of lipid ROS was significantly upregulated by treatment with 8HQ-Fe(III) and could be completely inhibited by Fer-1. Treatment with 8HQ-Fe(III) also increased the fluorescence intensity of Liperfluo, another LPO marker for detecting peroxyl radicals and lipid hydroperoxides [34], which was blocked by Fer-1 (Figure 3C,F). The reactive byproduct 4-hydroxynonenal (4-HNE), which resulted from the degradation of lipid peroxides, had been shown to contribute significantly to ferroptosis [50]. 8HQ-Fe(III) induced significant increases in 4-HNE content (Figure 3D).

Ferroptosis relies heavily on ROS generation and LPO, ultimately triggering morphological alterations in mitochondria (Mito) [51]. A key difference between ferroptosis and other types of cell death lies in the drastic morphological changes that take place in Mito during ferroptosis. In this study, transmission electron microscopy (TEM) at 4800× and 23,000× magnification indicated that 8HQ-Fe(III) induced significant mitochondrial swelling rather than mitochondrial shrinkage, which is a classic morphological feature of ferroptosis. (Figure 3G). Since 8HQ itself exhibits no biological effect at the experimental concentration, the 8HQ alone group is not shown in the experiments on intracellular iron concentration and lipid peroxidation.

Collectively, the above results demonstrated that 8HQ and Fe(III) formed a lipophilic iron complex that entered cells via simple diffusion, inducing lipid peroxidation and subsequent iron-dependent cell death. However, this cell death could not be inhibited by ferroptosis inhibitors and did not exhibit the morphological features of ferroptosis.

### 3.3. Mitochondrial Damage Played a Dominant Role in 8HQ-Fe(III)-Induced Cell Death, Rather than ER Peroxidation

**Figure 4:**
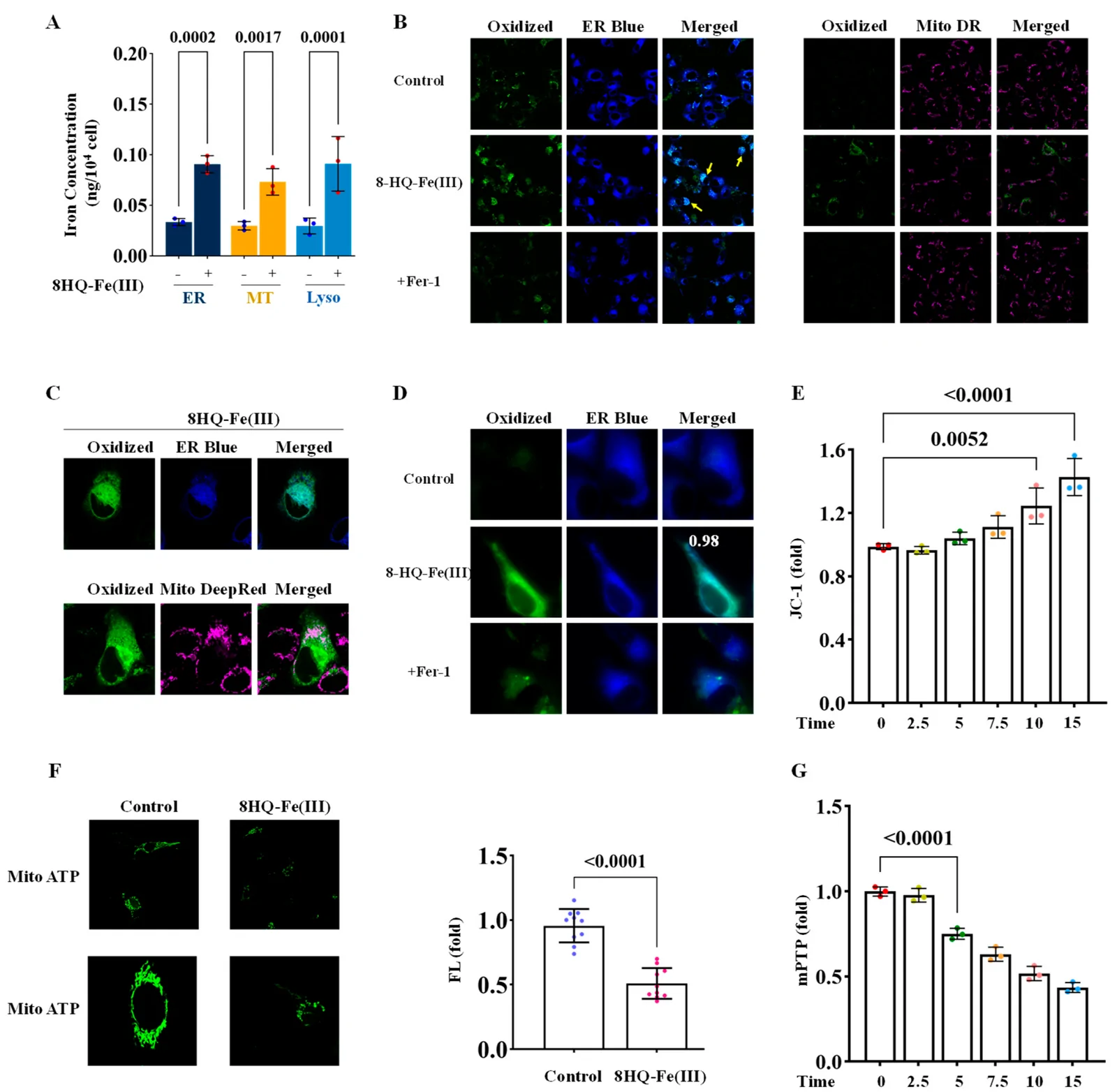
**ER peroxidation and Mito dysfunction induced by 8HQ-Fe(III).** (**A**) ICP-MS analysis of the iron concentration in different organelles, such as ER, Mito, and Lyso, under the treatment of 10 μM 8HQ-Fe(III) for 1 h. (**B**–**D**) Representative images of HT-1080 cells treated with 8HQ-Fe(III) for 30 min and stained with C11 BODIPY^581/591^ and ER tracker blue–white or Mito tracker Deep Red, as measured by CLSM (**B**,**C**) or SIM (**D**). (**E**) The change in JC-1 in HT-1080 cells under treatment with 10 μM 8HQ-Fe(III) for 2.5, 5, 7.5, 10, and 15 min, as measured by FACS. (**F**) The synthesis of ATP in HT-1080 cells under treatment with 10 μM 8HQ-Fe(III) for 30 min, using pCMV–Mito–AT1.03 as probe, as measured by CLSM. The quantified fluorescence data. (**G**) The change in MPTP opening in HT-1080 cells under the treatment of 10 μM 8HQ-Fe(III) for 2.5, 5, 7.5, 10, and 15 min, as measured by FACS. Control: untreated cells.

Given that 8HQ-Fe(III) induced cell death is dependent on iron, we further investigated the intracellular distribution of iron to explore the relationship between its subcellular localization and cell death. The iron concentrations in different organelles under treatment with 8HQ-Fe(III) were analyzed through ICP-MS (Figure 4A). In the 8HQ-Fe(III)-treated group, a notable elevation of iron concentrations was observed in the ER, Mito and lysosomes. As shown in Figure 4B,C, co-staining with C11 BODIPY581/591 and ER Tracker Blue-White revealed that 8HQ-Fe(III) induced ER peroxidation, which was markedly inhibited by Fer-1. 8HQ-Fe(III)-induced ER peroxidation was also confirmed through SIM with a higher spatial resolution (Figure 4D). The Pearson correlation coefficient was 0.98.

Considering that 8HQ-Fe also induced a significant increase in Mito iron level, Analysis of Mito dysfunction was further performed under the treatment of 8HQ-Fe(III). Mitochondrial membrane potential (MMP) served as a widely accepted marker for discerning Mito function [52]. The normal state of MMP is a prerequisite for sustaining mitochondrial oxidative phosphorylation, and its stability is advantageous in preserving normal cellular activities [52]. The Mito-staining dye JC-1 was used to study the Mito function. If JC-1 is added to cells with properly functioning Mito, it exists in its aggregated (red) state. By contrast, in compromised Mito, JC-1 assumed its monomeric (green) form as a result of MMP dissipation. 8HQ-Fe(III) significantly enhanced the green fluorescence of JC-1 in a time-dependent manner, indicating the dissipation of MMP (Figure 4E). 

The MPTP refers to a highly non-discriminatory channel located on the inner membrane of Mito that regulates their permeability and can contribute to cell death [53]. The opening of MPTPs is typically associated with various factors, including increased calcium ion concentration, loss of MMP, oxidative stress, and LPO [54]. As Figure 4G demonstrated, 8HQ-Fe(III) caused a significant decrease in the green fluorescence intensity, and this revealed a significant difference from the control group within only 5 min. This indicated that 8HQ-Fe(III) was able to strengthen or induce the opening of MPTP. Rauen et al. also suggested that the cell death induced by 8HQ-Fe(III) is closely tied to dysfunction in Mito permeability [40]. Treatment with 8HQ-Fe(III) also induced Mito swelling, suggesting that this chemical had the capacity to stimulate the opening of MPTP. Mitochondrial ATP synthesis is essential for preserving cell viability and function [55]. The loss of membrane potential and the opening of MPTP can lead to the dissipation of the proton gradient and subsequently inhibit the synthesis of ATP. In Figure 4F, 8HQ-Fe(III) could significantly inhibit the synthesis of ATP. These results strongly suggested that 8HQ-Fe(III) likely reduced MMP and induced the opening of MPTP to cause the dissipation of the proton gradient, and subsequently caused the inhibition of ATP synthesis and Mito dysfunction.

Although 8HQ-Fe(III) also induced lysosomal iron overload, it did not cause significant lysosomal damage. LysoTracker and acridine orange (AO) staining demonstrated no marked lysosomal damage (Figure S3).

**Figure 5:**
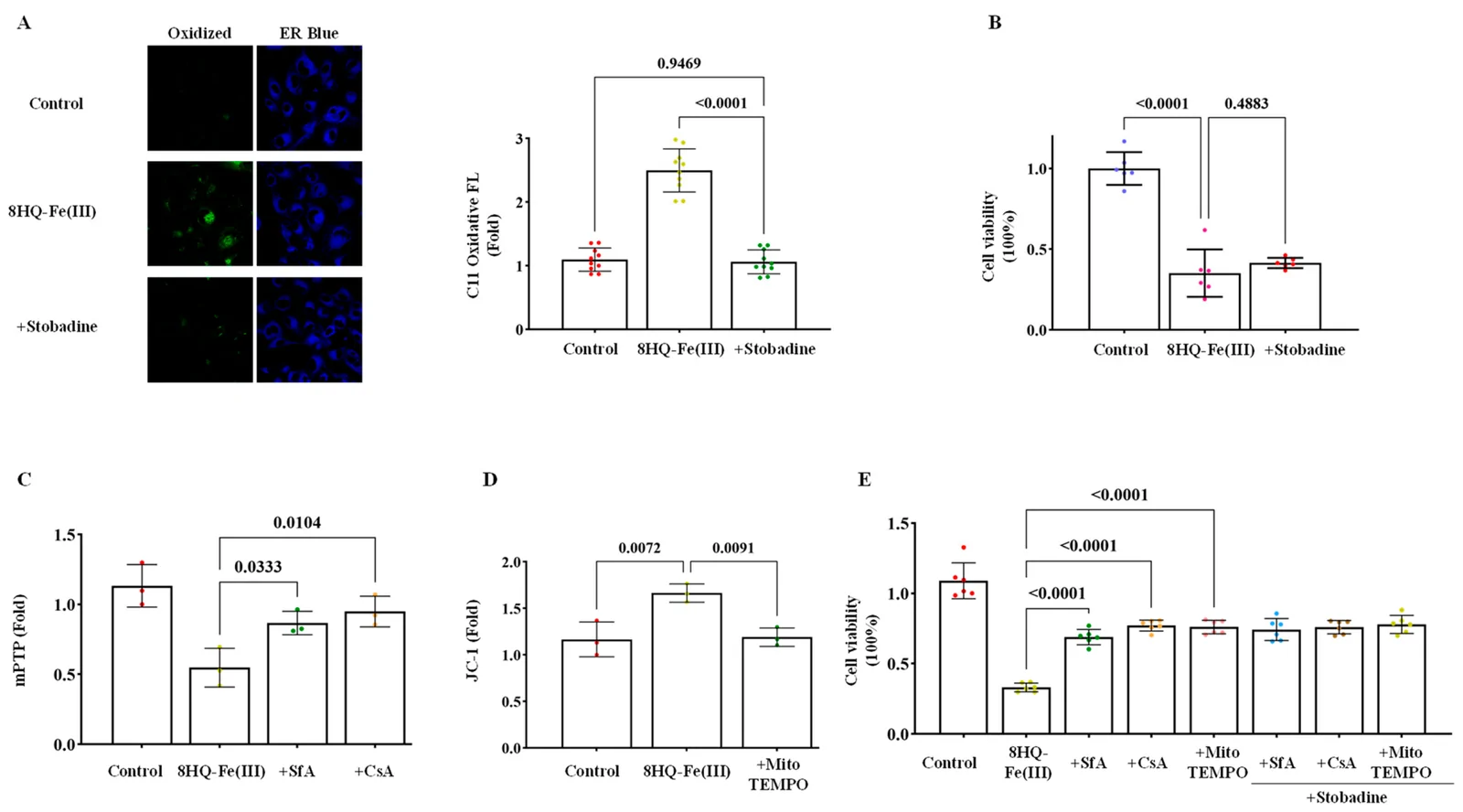
**The role of ER and Mito in 8HQ-Fe(III)-induced cell death.** (**A**) Representative images of HT-1080 cells treated with 8HQ-Fe(III) in the absence or presence of stobadine for 15 min and stained with C11 BODIPY^581/591^ and ER tracker blue-white. The quantified fluorescence data. (**B**) Rescue of HT-1080 cells treated with 8HQ-Fe(III) (10 μM, 1:1, 2 h) by stobadine. Data are represented as mean ± SD, n  =  6 biologically independent samples. (**C**) The change in MPTP opening in HT-1080 cells under treatment with 10 μM 8HQ-Fe(III) for 15 min, in the absence or presence of SfA or CsA, as measured by FACS. (**D**) The change in JC-1 in HT-1080 cells under treatment with 10 μM 8HQ-Fe(III) for 15 min, in the absence or presence of Mito TEMPO, as measured by FACS. (**E**) Rescue of HT-1080 cells treated with 8HQ-Fe(III) (10 μM, 1:1, 2 h) by SfA, CsA and Mito TEMPO. Control, untreated cells. Data are represented as mean ± SD, n  =  6 biologically independent samples.

Based on the above results, it was speculated that 8HQ-Fe(III)-induced cell death was closely associated with ER peroxidation and Mito damage. Therefore, we further investigated the critical roles of ER peroxidation and Mito damage in 8HQ-Fe(III)-induced cell death. In Figure 5A,B, treatment with the ER-targeted protective agent stobadine inhibited ER peroxidation but failed to suppress 8HQ-Fe(III)-induced cell death. Moreover, previous results also showed that Fer-1, despite inhibiting ER peroxidation, failed to prevent cell death. These findings indicated that ER peroxidation did not play a dominant role in 8F-induced cell death. Regarding Mito damage, treatment with the Mito mPTP protectants SfA or CsA significantly suppressed 8HQ-Fe(III)-induced mPTP opening (Figure 5C). MitoTEMPO also inhibited the dissipation of MMP induced by 8HQ-Fe(III) (Figure 5D). More interestingly, SfA, CsA or MitoTEMPO also significantly blocked 8HQ-Fe(III)-induced cell death (Figure 5E), indicating that 8HQ-Fe(III)-induced cell death was closely associated with Mito damage, but not ER peroxidation.

As is known from previous studies, ER peroxidation played an important role in ferroptosis. The failure of 8HQ-Fe(III)-induced cell death to exhibit ferroptotic characteristics may be closely related to the fact that ER peroxidation did not play a key role in this process.

### 3.4. DHA Converted 8HQ-Fe(III)-Induced Non-Ferroptosis into Ferroptosis by Enhancing the Role of ER Peroxidation in Cell Death

**Figure 6:**
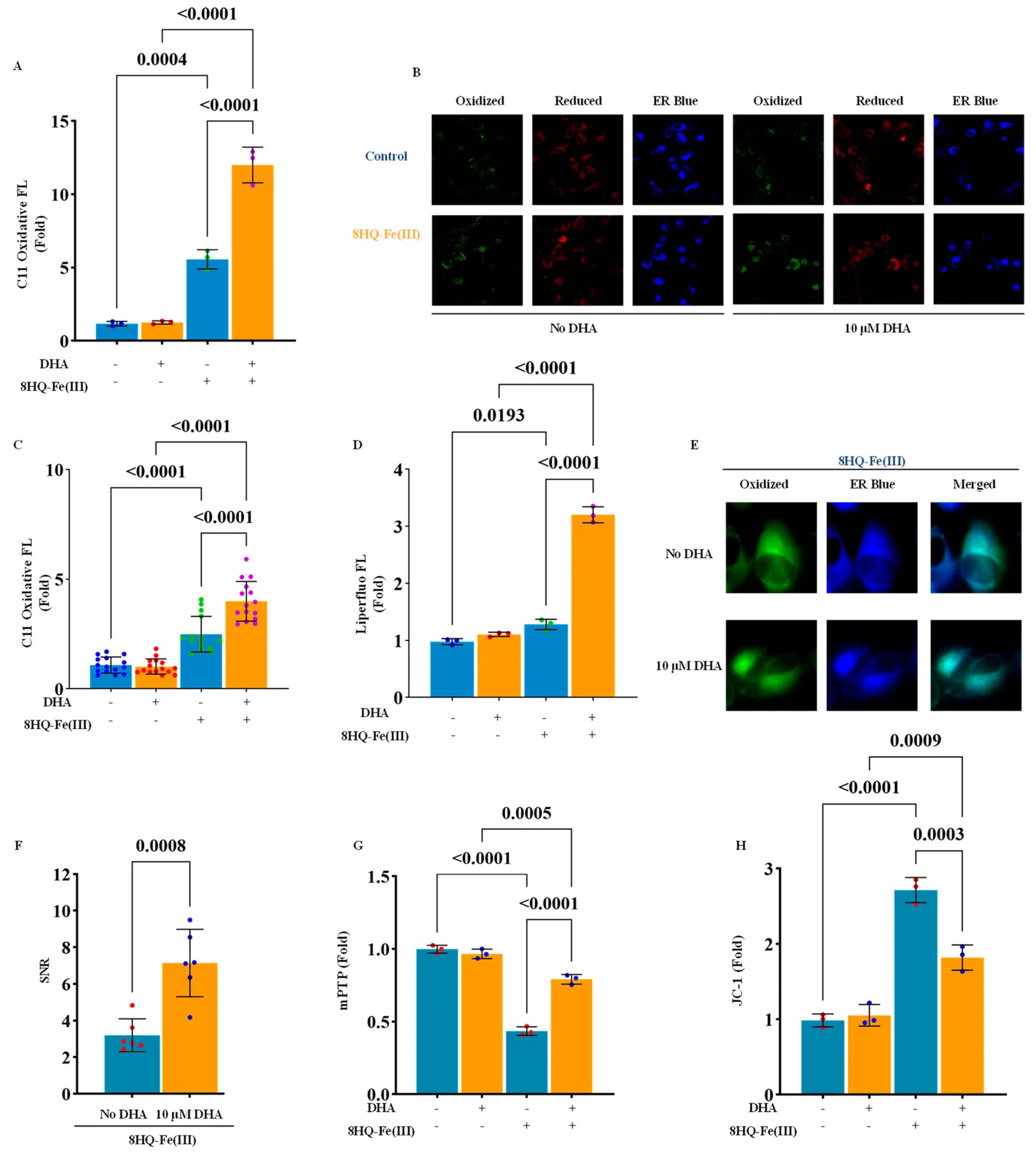
**Preincubation with DHA enhanced ER peroxidation and alleviated Mito dysfunction induced by 8HQ-Fe(III).** (**A**) Effect of pre-treatment (24 h) with DHA on LPO induced by 10 μM 8HQ-Fe(III) for 15 min in HT-1080 cells, using C11 BODIPY^581/591^ as probe, as measured by FACS. Data are represented as mean ± SD, n  =  3. (**B**) Representative confocal microscopy images showing the effect of pre-treatment (24 h) with DHA on LPO in the cells treated with 8HQ-Fe(III), respectively, as reflected by C11 BODIPY^581/591^ probes. Quantified data of cellular fluorescence were displayed in (**C**), with the values represented as the mean ± SD, n  =  15. (**D**) Effect of pre-treatment (24 h) with DHA on LPO induced by 10 μM 8HQ-Fe(III) for 15 min in HT-1080 cells, using Liperfluo as probe, as measured by FACS. Data are represented as mean ± SD, n  =  3. (**E**) Representative SIM images showing the effect of pre-treatment (24 h) with DHA on LPO triggered by 10 μM 8HQ-Fe(III) for 15 min in HT-1080 cells, using C11 BODIPY^581/591^ as probe, co-stained with ER tracker blue-white. Quantified data of signal/noise ratio (SNR) are shown in (**F**). Data are represented as mean ± SD, n  = 6. (**G**) Effect of pre-treatment (24 h) with DHA on MPTP opening induced by 10 μM 8HQ-Fe(III) for 15 min in HT-1080 cells. (**H**) Effect of pre-treatment (24 h) with DHA on JC-1 induced by 10 μM 8HQ-Fe(III) for 15 min in HT-1080 cells. Control, untreated cells.

The ER is the largest membranous organelle in eukaryotic cells, occupying approximately half of the cellular membrane system. The ER synthesizes nearly all membrane lipids required by eukaryotic cells, including phospholipids and cholesterol [56,57]. Polyunsaturated fatty acids (PUFAs), important substrates for phospholipid synthesis, are concentrated within the ER membrane [58]. Recent studies have also indicated that PUFAs predominantly integrate into phospholipids within this organelle [36,59]. Certain ω-3 PUFAs, such as eicosapentaenoic acid (EPA) and docosahexaenoic acid (DHA), have high numbers of double bonds, which makes them potentially more prone to oxidation than linoleic acid, arachidonic acid, and adrenic acid. DHA is prominently present in the brain, retina, and sperm, and all of these are susceptible to ferroptosis [59,60]. Previous studies have indicated that DHA can enhance ER peroxidation by increasing ER abundance [36]. Thus, in this study, DHA was used to investigate changes in cell death triggered by 8HQ-Fe(III). Changes in LPO were evaluated following pre-incubation with DHA. As expected, DHA significantly increased the LPO induced by 8HQ-Fe(III) (Figure 6A–D). As shown in Figure 6A–C, DHA pre-incubation significantly enhanced the fluorescence of the oxidized form of C11 BODIPY^581/591^ induced by 8HQ-Fe(III). Another LPO probe, Liperfluo, was also used to show that DHA pre-incubation significantly increased the LPO induced by 8HQ-Fe(III) (Figure 6D). Furthermore, upon co-staining with C11 BODIPY^581/591^ and ER tracker blue-white, DHA enhanced ER peroxidation via structured illumination microscopy (SIM) (Figure 6E,F). Preincubation with DHA also attenuated 8HQ-Fe(III)-induced mitochondrial damage (Figure 6G,H). Recent investigations indicate that DHA supplementation may potentially lower the vulnerability of heart mitochondria to permeability transition, a detrimental process that frequently results in cell death [61]. DHA deficiency may also lead to a decrease in MMP in hepatocytes [62]. DHA alone (10 μM, 24 h) showed no significant effect on LPO and mitochondrial damage.

**Figure 7:**
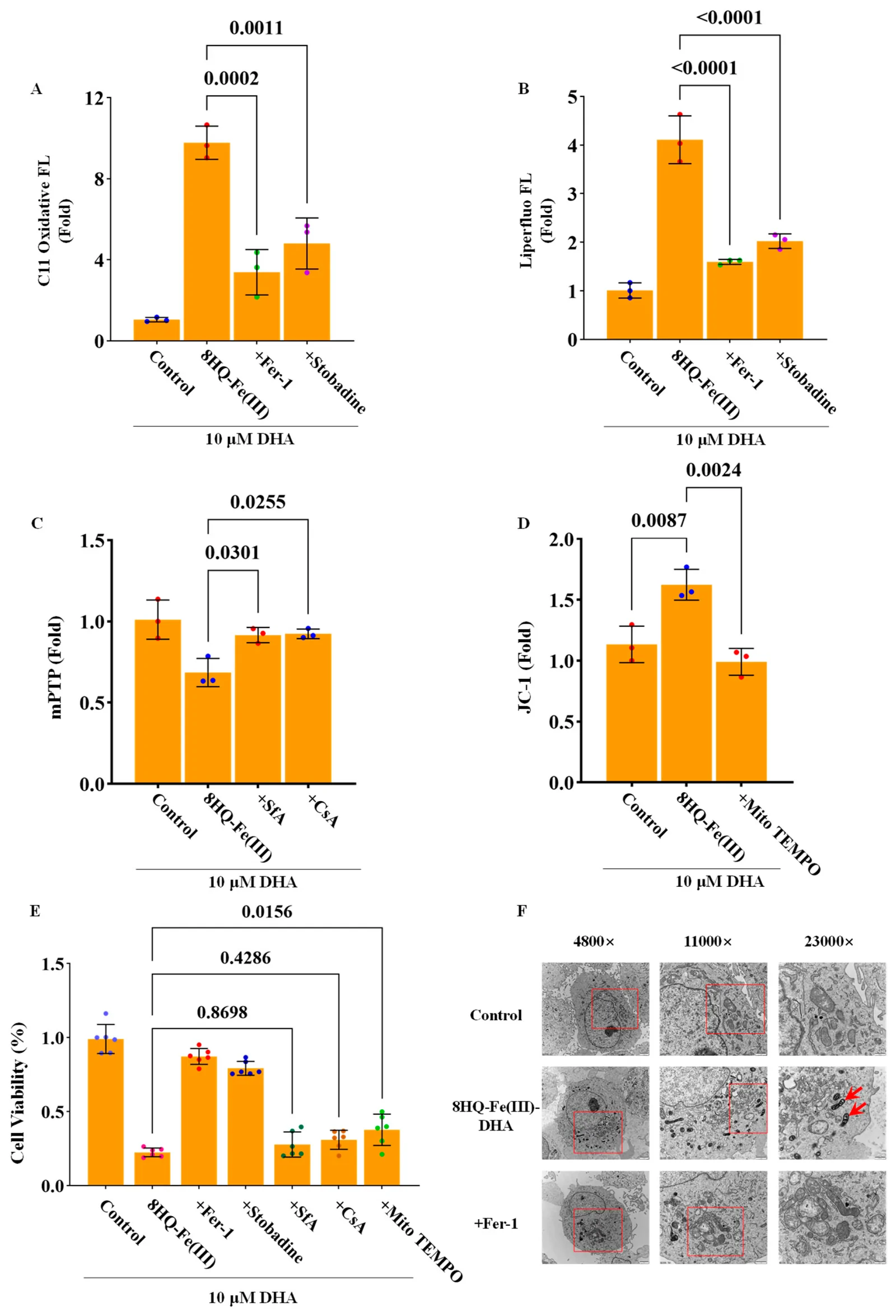
**Preincubation with DHA alters cell death induced by 8HQ-Fe(III).** (**A**,**B**) Under DHA pre-treatment, the change in LPO in HT-1080 cells treated with 8HQ-Fe(III) (10 μM, 1:1, 15 min) in the absence or presence of stobadine or Fer-1, and stained with C11 BODIPY^581/591^ (**A**) or Liperfluo (**B**), as measured by FACS. (**C**) Under DHA pre-treatment, the change in MPTP opening in HT-1080 cells under treatment with 10 μM 8HQ-Fe(III) for 15 min, in the absence or presence of SfA or CsA, as measured by FACS. (**D**) Under DHA pre-treatment, the change in JC-1 in HT-1080 cells after treatment with 10 μM 8HQ-Fe(III) for 15 min, in the absence or presence of Mito TEMPO, as measured by FACS. (**E**) Under DHA pre-treatment, rescue of HT-1080 cells treated with 8HQ-Fe(III) (10 μM, 1:1, 1 h) by Fer-1, stobadine, SfA, CsA and Mito TEMPO. Data are represented as mean ± SD, n  =  6 biologically independent samples. (**F**) Under the pre-treatment of DHA, TEM of HT-1080 cells treated with 8HQ-Fe(III) (10 μM), in the absence or presence of Fer-1 (10 μM). Red arrow, damaged mitochondria. The untreated control data are shown in Figure 6 and Figure 1.

Furthermore, under DHA pre-incubation conditions, the ER peroxidation induced by 8HQ-Fe(III) was suppressed by stobadine and Fer-1 (Figure 7A,B), and the Mito damage induced by 8HQ-Fe(III) was also inhibited by SfA, CsA, and MitoTEMPO (Figure 7C,D). More strikingly, contrary to the results without DHA pre-incubation (Figure 1A and Figure 5B), after DHA pre-incubation, 8HQ-Fe(III)-induced cell death was significantly inhibited by Fer-1 and stobadine, whereas Mito protectors failed to exert any significant inhibitory effect on 8HQ-Fe(III)-induced cell death (Figure 7E). Furthermore, the Mito exhibited the morphological characteristics of ferroptosis, namely Mito shrinkage (Figure 7F). DHA alone (10 μM, 24 h) showed no significant cytotoxicity. 

To confirm that the switch in cell death mode is not specific to HT-1080 cells, we performed similar experiments in AML12 cells. Without DHA pretreatment, neither Fer-1 nor stobadine rescued 8HQ-Fe(III)-induced cell death. In contrast, after DHA pretreatment, both Fer-1 and stobadine significantly inhibited cell death. These results are consistent with our findings in HT-1080 cells, indicating that DHA switches 8HQ-Fe(III)-induced non-ferroptotic death to ferroptosis in a cell line-independent manner (Figure S4).

**Figure 8:**
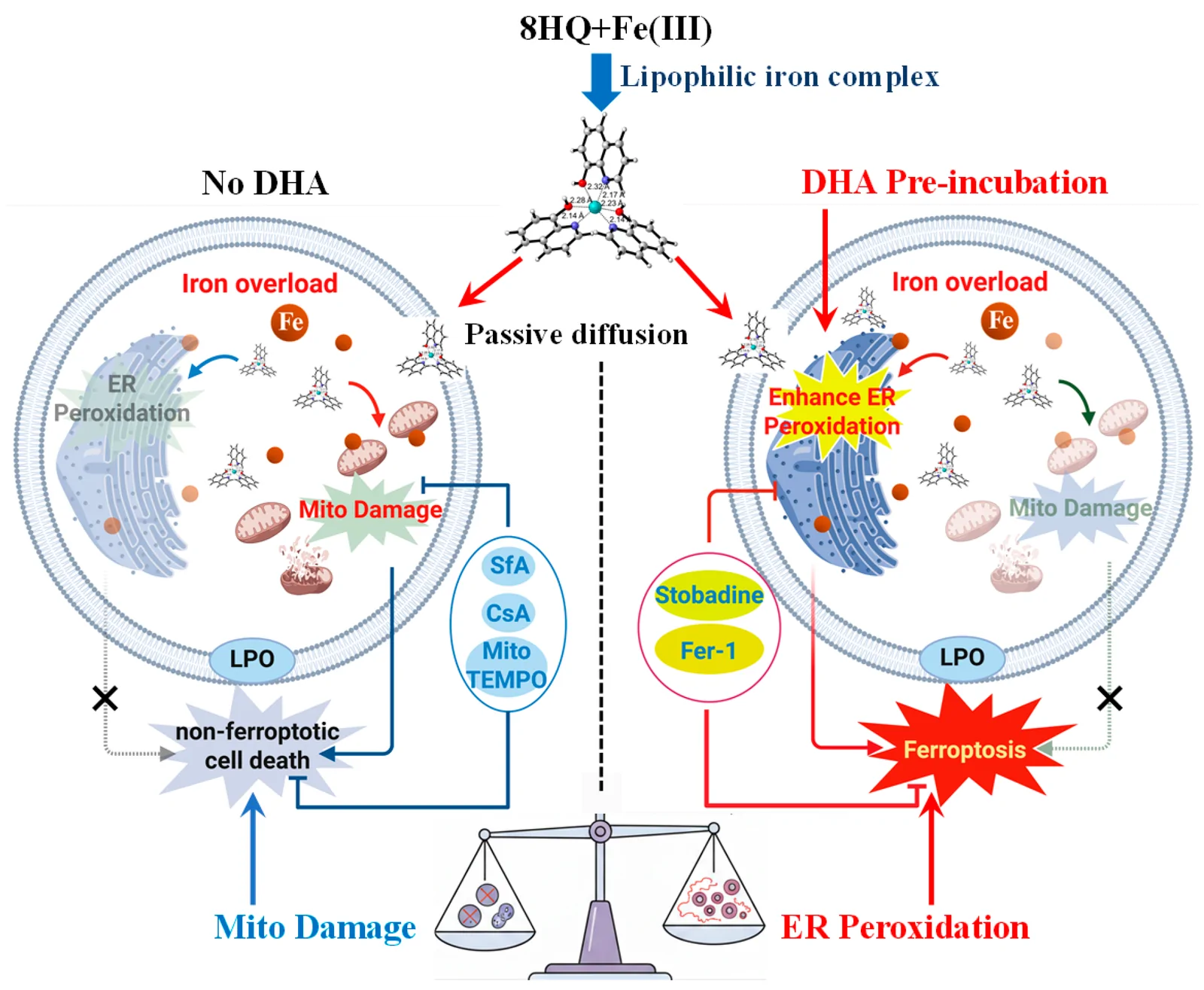
**Proposed mechanism of ferroptosis induced by 8HQ-Fe(III) combined with DHA in HT-1080 cells.**

This suggested that DHA can convert 8HQ-Fe(III)-induced “iron-dependent non-ferroptosis” into classical ferroptosis through the dual regulation of “enhancing ER peroxidation and inhibiting mitochondrial damage”. These results further indicated that the balance between ER peroxidation and mitochondrial damage may be the key factor determining the type of iron-dependent cell death. Therefore, by enhancing ER peroxidation and making it the dominant driver in cell death, DHA converted iron overload-induced non-programmed cell death into ferroptosis (Figure 8).

## 4. Discussion

A number of studies have provided compelling evidence linking ferroptosis to multiple disorders, with iron overload identified as a key contributing factor. The development of highly efficient ferroptosis inducers and the establishment of in cellulo and in vivo disease models that simulate iron overload-induced ferroptosis are important for the treatment of such diseases [11,12,13,14,16,63]. Selecting highly efficient iron chelators that can effectively transport iron into cells by forming lipophilic complexes and thus inducing iron overload was a promising strategy of research. However, this field remained underexplored. It was likely that the cell death induced by lipophilic iron complexes via iron overloading did not always display classical ferroptosis characteristics, and the underlying mechanisms still remained largely unclear.

In the present study, it was suggested that the key reason why 8HQ-Fe(III) failed to induce ferroptosis through iron overload was that ER peroxidation did not play a dominant role in this cell death; instead, mitochondrial damage played the primary role. Notably, DHA can convert 8HQ-Fe(III)-induced “iron-dependent non-ferroptosis” into classical ferroptosis by dual regulation: enhancing ER peroxidation and inhibiting mitochondrial damage. 

These results further indicated that the balance between ER peroxidation and mitochondrial damage may be the key factor determining the type of iron-dependent cell death. Therefore, by enhancing ER peroxidation and making it the dominant driver of cell death, DHA converted iron overload-induced non-programmed cell death into ferroptosis. These findings provided proof-of-concept evidence that the mode of iron-dependent cell death can be readily switched by manipulating the subcellular balance of peroxidation and organelle damage.

Compared to conventional ferroptosis inducers (e.g., erastin, RSL3, and FINO_2_), the 8HQ-Fe(III) system combined with DHA offered several unique advantages: (1) it achieved rapid iron delivery and LPO within hours at low concentrations; (2) the combination of a simple iron chelator (8HQ) with a natural DHA was readily accessible and cost-effective; and (3) it allowed precise control over the ferroptotic pathway by modulating ER peroxidation, a subcellular compartment increasingly recognized as a central hub for ferroptosis execution. Recent studies have highlighted that the ER membrane was a primary site for lethal LPO [36], and our results supported the notion that if iron overload-induced ER peroxidation was the primary cause of cell death, then this cell death was likely to be ferroptosis. This may represent a more efficient and mechanistically defined approach to induce ferroptosis.

The ER serves as a pivotal orchestrator of peroxidation-driven cell death. Previous studies have indicated that ferroptosis is closely associated with ER peroxidation [36,64]. Our present data suggest that pre-incubation with DHA, which shifted iron overload-induced ER peroxidation to the major driver of cell death, could serve as a general strategy for triggering ferroptosis in a controlled manner. Moreover, given that 8HQ-Fe(III) alone induced mitochondrial-dominated non-ferroptotic death, this system also provided a valuable tool to dissect the interplay between organelle-specific iron accumulation and cell death modality. In future studies, exploring the temporal relationship between ER peroxidation and mitochondrial damage may further clarify how the mode of iron-dependent cell death is determined. These studies may also extend this approach to in vivo models, potentially enabling the development of novel ferroptosis-based therapies for diseases characterized by iron dyshomeostasis and ER peroxidation. For such translation, the bioavailability of 8HQ-Fe(III) and DHA is an important consideration. 8HQ-Fe(III) is a lipophilic small-molecule complex that enters cells by passive diffusion, and DHA is a natural ω-3 fatty acid with good biocompatibility. Their combination may be suitable for local or systemic administration. However, in vivo use still needs to consider iron overload risk, DHA dosage, tissue targeting, and pharmacokinetics. Nanoparticle delivery and targeted prodrug strategies may help address these issues. Safety and efficacy should also be evaluated in animal models before eventual human translation. While the mechanistic experiments were mainly performed in HT-1080 cells, key phenotypes were confirmed in AML12 cells, indicating that the observed switch in cell death mode is not specific to HT-1080. Nevertheless, further studies using additional cell lines, including normal cells, are needed to evaluate the broader applicability and translational potential of our findings.

## Data Availability

The data supporting this article are presented within the main text and the Supplementary Information.

## Supplementary Materials

The following supporting information can be downloaded at: https://www.mdpi.com/article/10.3390/antiox15101303/s1, Figure S1. Inhibitory effect of apoptosis or necroptosis inhibitors on the cell death induced by 8HQ-Fe(III). Figure S2. ESR spectra produced by incubation of DMPO with Fe(III), with or without DMSO. Figure S3. A. Representative images of HT-1080 cells treated with 8HQ-Fe(III) for 15 min and stained with LysoTracker DR, as measured by CLSM. B. The mean fluorescence intensity of green fluorescence in the AO relocation experiment. Figure S4. Rescue of AML12 cells treated with 8HQ-Fe(III) (10 μM, 1:1, 1 hour) by Fer-1, stobadine, with or without DHA pretreatment.
