# Supplementary material for: Unusual Switch of Cell Death Mode by Docosahexaenoic Acid (DHA) via Enhancing Endoplasmic Reticulum Peroxidation Induced by Lipophilic Iron Complex
Source: Antioxidants (Basel). 2026 Oct 9;15(10):1303. doi: 10.3390/antiox15101303 (PMC13700758; doi:10.3390/antiox15101303)
Supplement: Supplementary file 1 [file antioxidants-15-01303-s001.zip › antioxidants-4530290-supplementary.pdf]

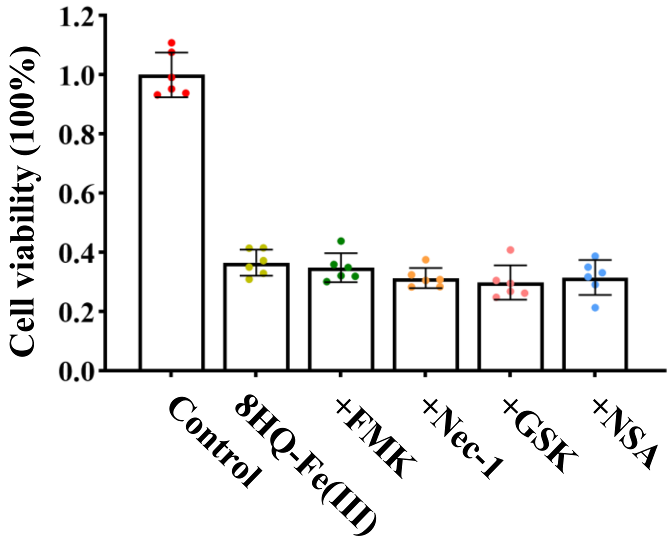

Figure S1. Inhibitory effect of apoptosis or necroptosis inhibitors, such as 50 μM Z-VAD-FMK (FMK), 10 μM Necrostatin-1 (Nec-1), 3 μM GSK872 (GSK) and 3 μM Necrosulfonamide (NSA) on the cell death induced by 8HQ-Fe(III).

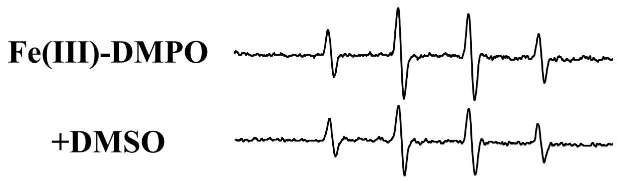

Figure S2. ESR spectra produced by incubation of DMPO with Fe(III), with or without DMSO.

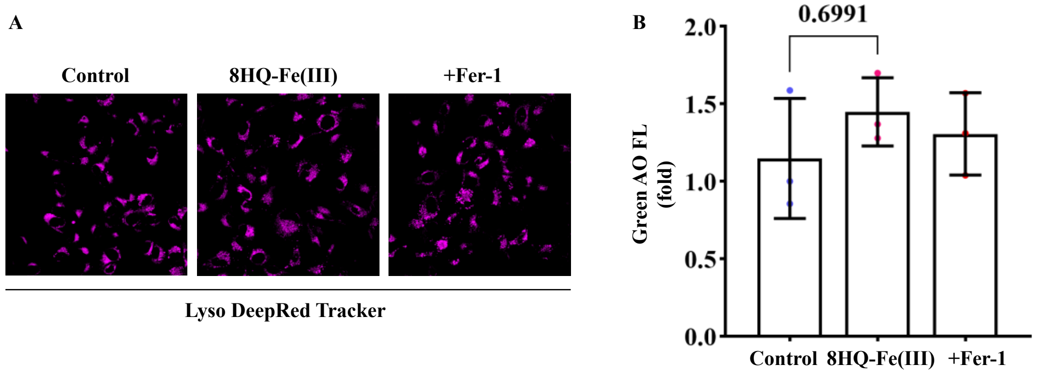

Figure S3. A. Representative images of HT-1080 cells treated with 8HQ-Fe(III) for 15 min and stained with Lyso tracker DR, as measured by CLSM. **B**. The mean fluorescence intensity of green fluorescence in the AO relocation experiment. Data are represented as mean ± SD, n = 3.

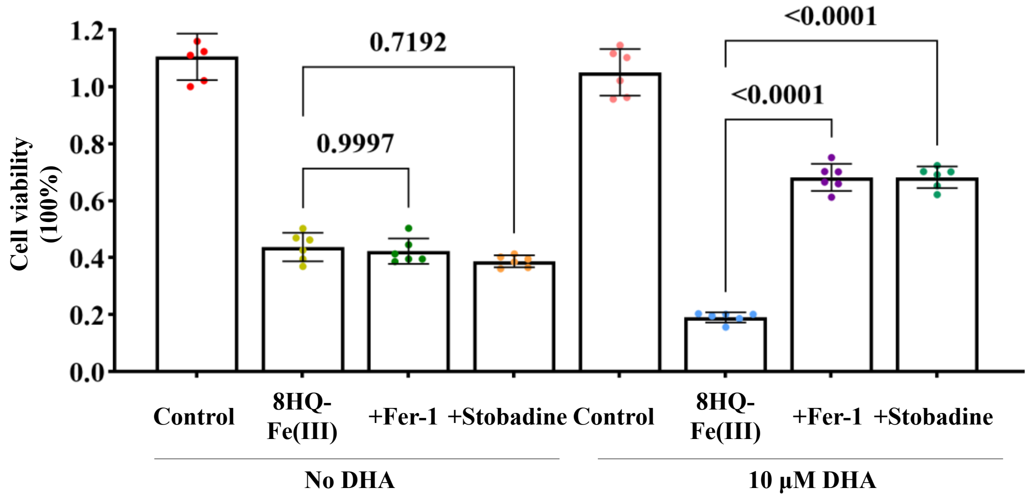

Figure S4. Rescue of AML12 cells treated with 8HQ-Fe(III) (10 μM, 1:1, 1 hour) by Fer-1, stobadine, with or without DHA pretreatment. Data are represented as mean ± SD, n = 6 biologically independent samples.
